# System, Space, Staff, and Stuff framework in establishing a new pediatric critical care unit (PICU) (4S Framework)

**DOI:** 10.26502/jppch.74050129

**Published:** 2022-10-12

**Authors:** Hakem Alomani, Fawaz Alanzi, Yousef Alotaibi

**Affiliations:** 1 Pediatric and Pediatric critical care Consultant, Pediatric Department, Pediatric Critical Care Section, King Faisal Specialist Hospital & Research Center, Madinah, Saudi Arabia; 2 Assistant professor, College of Medicine, Alfaisal University, Consultant, Pediatric Critical Care Section, Pediatric Department, King Faisal Specialist Hospital & Research Center, Riyadh, Saudi Arabia; 3 Pediatric Intensivist, Children Hospital, King Saudi Medical City, Riyadh KSA

**Keywords:** PICU, design of PICU, renovating, staff, staff, space, framework, establishment of PICU, healing environment

## Abstract

By virtue of being in a developing country with ongoing expanding of the healthcare system, establishing or at least renovating a Pediatric critical care unit (PICU) has become a necessity. As intensivists and healthcare providers, we excel at our job as clinicians; however, we perform less than perfect when it comes to participating in establishing new PICUs and deliberately building and designing an EBM and patient-centered PICU with a complete understanding of the technical and non-clinical processes during commissioning or operational phases like construction, physical layout (blueprint), Biomedical engineering aspects, equipment, supply, and work-environment enhancement. If all healthcare providers -and especially intensivists- avoid being involved actively in PICUs designing process at their institution, they will miss an opportunity to gain a new perspective as well as they might contribute to a fragmented process of ICU design and a suboptimal result that might impact the PICU environment, patient journey and eventually the quality of care in that ICU.

The PICU designing processes should be handled via a multi-professional team approach in an integrated -not parallel- manner that includes clinical and non-clinical personnel. Therefore, the processes will be more integrated, and they will finish the project efficiently, effectively, safely, and patient-centered way.

This paper is an expert opinion and literature review that describes a conceptual framework to guide simple and practical mental processes in establishing and designing processes for new PICUs in developing countries. We called this preparedness tool: the 4S framework (system, space, staff, and stuff). It is a well-known preparedness tool that is commonly used in planning new projects by project leaders. Therefore, we utilized it in establishing a new PICU intended to meet the national and international accreditation standards and requirements. This unique preparedness tool will help establish an easy conceptual framework for all healthcare providers to grasp the complex -clinical and non-clinical- processes of establishing new PICUs and develop a holistic approach to this complex project.

Note: The authors had leading roles in establishing or renovating many PICUs in Saudi Arabia, in both private and governmental hospitals, and would like to share their novel conceptual framework for establishing new PICUs in developing countries.

## Introduction

PICU is relatively a young subspecialty compared to adult ICU or even Neonatal ICU. In the USA, It was recognized as a pediatric subspecialty in 1985, and its first graduate pediatric intensivist certified with pediatric critical care was only in 1990 [1]. The concept of a closed PICU system and different PICU levels were established in the North American guidelines outlined in 1993 [2]. The level (I) PICU must provide the definitive care for variable complex and rapidly progressively medical, surgical, and traumatic disorders in the pediatric population. It should be located in major medical centers or within children’s hospitals. Ideally, the level (I) PICU should be able to provide care to the most critically ill patient population. Level (I) PICUs usually vary in size, personnel, physical layout, and types of equipment, in addition to the difference in the types of specialized care available, such as transplantation or cardiac surgery. (see supplement 1 for detailed requirements for Level I, II PICUs).

Level (II) PICUs should care for pediatric patients with moderate severity of illness and transfer more complex patients to Level I PICU for definite management. The requirements for level (II) PICUs are essentially differing from the criteria for level (I) PICUs in areas concerning the type and time required for physical presence in addition to hospital resources. Usually, level (II) PICU does not require the presence of the full spectrum of subspecialists. However, For every level (II) PICU, it is a must to have a robust communications system with a level (I) PICU to facilitate a timely referral of patients who need advanced care that is not established in the level (II) PICU. It’s essential to recognize that the duplication of services may lead to underutilization of resources, inadequate clinical personnel skills, and unfavorable cost-benefit analysis [3]. Regionalization of pediatric critical care -one large referral PICU in the region as opposed to too many small PICUS operating independently- is a cost-effective way to utilize the critical care services given the scarcity of qualified PICU staff [4].

Building an ICU with an Optimal design can help reduce medical errors, improve patient outcomes, reduce the length of stay, increase social support for patients, and reduce costs [5,6]. However, Optimal design requires knowledge of best practices, design standards, and building codes; hence engineers and non-clinicians are an integral part of the designing team [7,8].

In recognizing the impact of ICU design on the overall quality of care, the Society of Critical Care Medicine (SCCM), the American Association of Critical-Care Nurses (AACCN), and the American Institute of Architects/Academy of Architecture for Health (AIA/AAH) launched an annual ICU design competition (since 1993) to award the best design internationally [9,10]. However, most of the literature about ICU designs are adult based ICUs, and despite the considerable similarity in design between adult ICU and PICU, we might say: (PICU is NOT a small adult ICU), moreover, designing PICU is more challenging given the inherent heterogeneity in its population, i.e. accommodating tiny neonates, infants, children as well as adolescences with adult weight. All this calls for flexibility in design and attention to detail to create a healing environment for children.

Finally, all healthcare institutions need to meet the national and international accreditation standards to be recognized as high standard, safe and qualified facilities. In Saudi Arabia, we have our national accreditation body which is called the Saudi Central Board for Accreditation of Healthcare Institutions (CBAHI). It was established in 2005 as a not-for-profit organization under the umbrella of the ministry of health (MOH), and it is the only national agency with authority to grant accreditation certificates to any governmental or private healthcare facilities operating in Saudi Arabia where it’s a prerequisite document mandated by the government [20]. When a health care facility establishes a new PICU, it should take into consideration the national and international accreditation standards and implement these requirements in the design processes.

## Disclaimer

It is not the intent of this review to neither give an exhaustive list of all requirements and pre-requisites for building a new PICU nor supersede any established regulations or guidelines. However, the intent is to introduce a new conceptual framework to help clinicians navigate the PICU design process with clear goals without being lost in details.

## First

### Create a multidisciplinary team

The building or Establishing a new PICU is not one person’s job; instead, it’s a collective effort by different minds that gather and exchange their unique point of view in an integrated manner to paint a clear picture of a healing environment. This team or task force should -at a minimum- include a pediatric critical care physician, R.T., R.N., clinical engineers, project manager, Biomedical supply resource, information technology expert, infection control staff, Quality representative, safety officer, and an admin representative.

## Second

### Formulate a conceptual framework to guide the thought process

**Figure 1:**
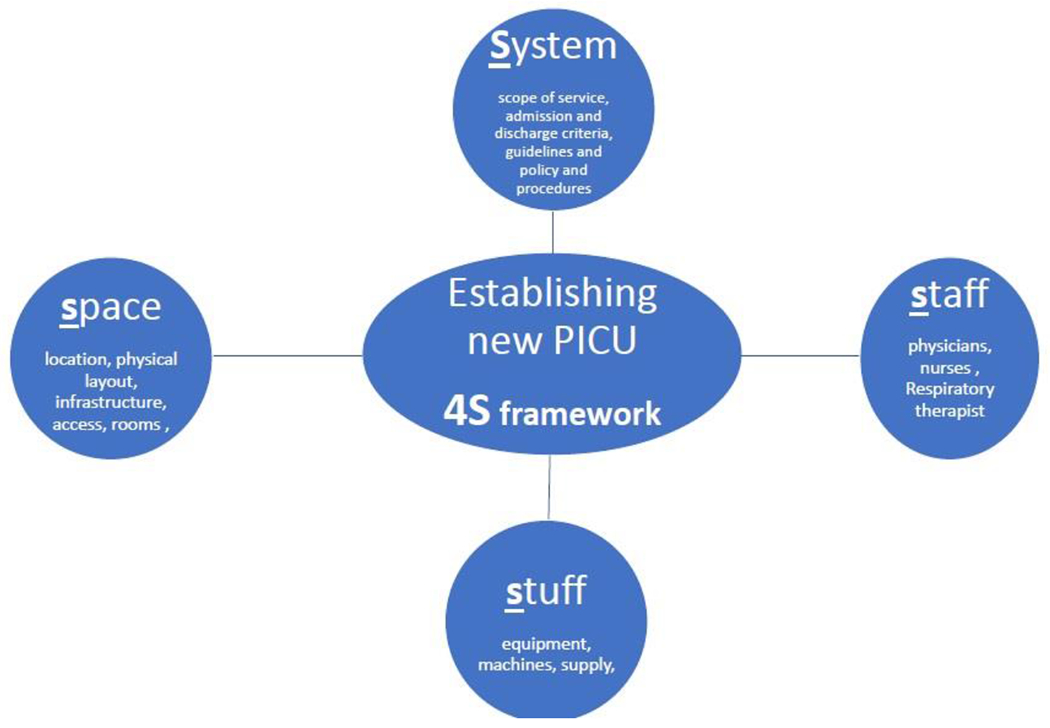
4S Framework.

Building a new PICU is a complex process and time-consuming endeavor. Without proper planning and a clear framework, the team will be overwhelmed with multiple competing interests and sometimes conflicting interests. We utilized and modified a common preparedness tool and used it as a conceptual framework we call it 4S (number four AND capital letter S) composed of system, space, staff, and stuff. where each (S) represents a distinct entity of design with different scope and requirements (Figure 1), (Table 1 cheat-sheet).

## System

The system here means the rules and policies governing the flow of work in the PICU. It’s advisable to create a folder with all system documents in one place. It’s mandatory to have a written organization chart -for system orientation as well as for the national and international accreditation requirements. It should delineate the line of authority starting from the individual PICU staff up to the higher chain of command in a flow diagram. Type of PCIU (open Vs. closed system) and Scope of service are essential documents to be written. Most modern PICUs are closed system PICU (covered by qualified intensivists 24/7); however, some small community PICUS are still partially covered by intensivists hence called open-system PICU. In the scope of service, we need to clarify the type of patient we plan to serve, including the age limit; moreover, our scope of service should align with our hospital’s overall vision and mission. We need to write clear admission and discharge criteria based on prespecified clinical or physiological parameters. A written job description with delineation of responsibilities inside PICU is a vital document to be clearly written to accommodate for different levels and competencies among PICU staff, i.e., PICU attending consultant Vs. PICU specialist or PICU resident. It’s preferable to create a protocol for any common process in PICU in order to standardize the work, i.e., sedation protocol, weaning from mechanical ventilation protocol, and feeding protocol. The presence of family in PICU is known to contribute to important patient outcomes via social interaction that is essential for a healing environment in critical care [11], hence guidelines for visitation must be agreed upon and written to include the time for visiting in PICU (preferably 24/7 for parents). Of note, parents’ staying (sleepover) in a designated bed inside PICU reduces parental stress in PICU and improves patient/parent experience [12].

## Space

**Figure 2:**
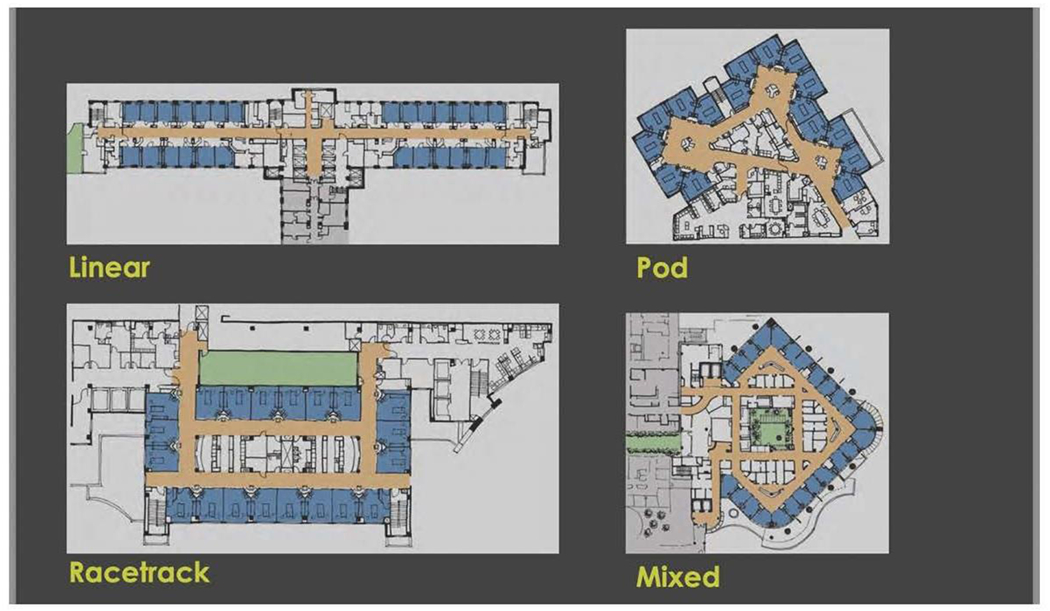
Types of Unit Configurations (Courtesy of Charles D. Cadenhead, FAIA, FACHA, FCCM Senior Principal, WHR Architects) (WHR ARCHITECTS).

**Figure 3:**
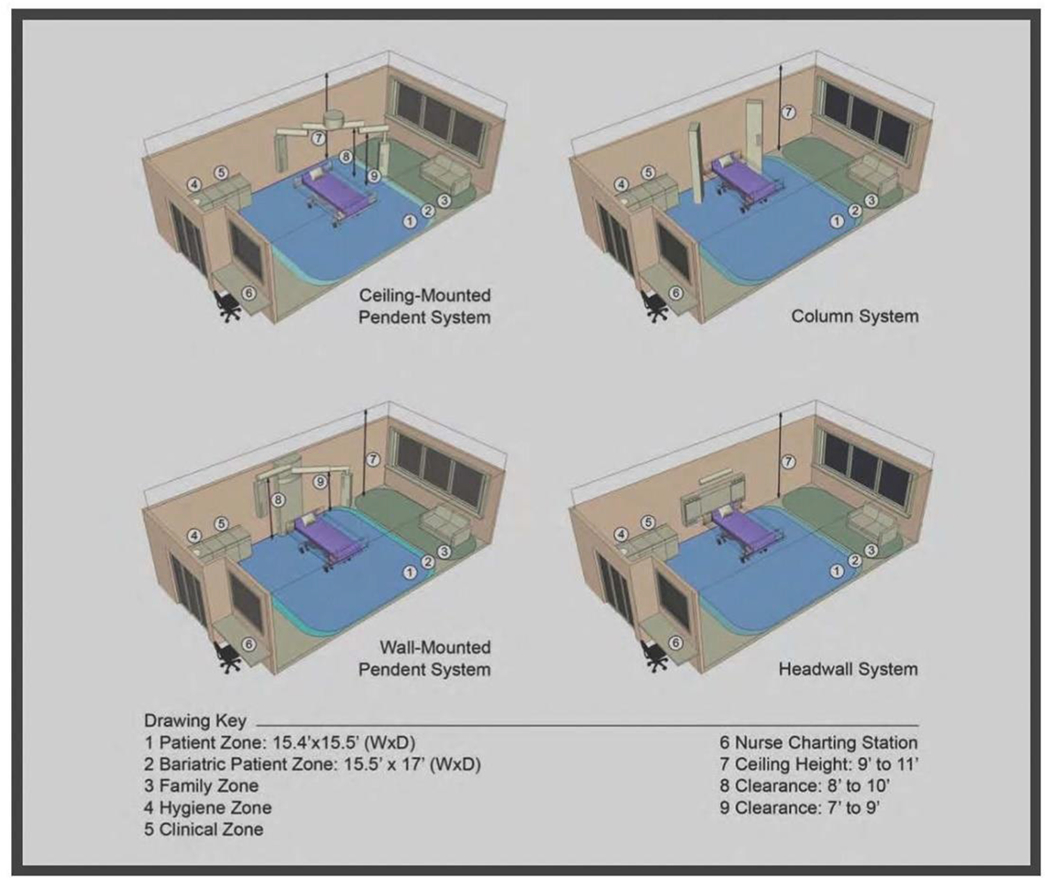
Different designs of medical gases and electrical outlets in ICU (Courtesy of Charles D. Cadenhead, FAIA, FACHA, FCCM Senior Principal, WHR Architects) (WHR ARCHITECTS).

Space here means the unit’s physical layout, including infrastructures (like medical gas supply, electricity) and the structure itself (like rooms, walls, windows). The location of the PICU must be discussed before starting the building process if that is possible. It’s imperative that PICU is located near the emergency department (ED) and operation theater (OR) with designated access to minimize traffic and expedite transport. The number of beds is a very challenging component that should be discussed thoroughly and planned based on the type of PICU (tertiary, secondary, community), the vision and mission of the hospital, and the available manpower. Overall, PICU with less than six beds risks insufficiency (waste of resources), and more than 14 become difficult to control under one unit (observation, noise control, traffic control). Therefore some experts recommend if more than 14 beds are needed, to decentralized the unit by Creating pods of 12-14 beds with different teams (Figure 2) [13]. The unit size and the bed number are known decisions that are linked to essential patients, staff, and financial outcomes; hence, a decision matrix was proposed to strike a balance between positive patient experience, staff experience, quality, and a financial burden [14]. Rooms in PICU can be divided into patient rooms, clinical rooms, and non-clinical rooms. The patient’s room is the core site for all the actions; thus, it needs to be spacious, easy to access, and easy to monitor. Ideally, the patient room is a single room design with head-wall mounted medical gases or a ceiling-mounted pendant (Figure 3). Single room design increases privacy and might decrease nosocomial rate infection in PICU and is considered superior to a multi-beds room [15,16].

Evidence suggests that electrical power, monitoring outlets, and medical gases with a ceiling-mounted design are better than the traditional head-wall mounted in term of team dynamic and accessibility; however, there is an extra financial cost for this delicate modern design that need to be balanced cost-effectively with the available resources [17]. Ideally, this single room should have a sliding glass door to enhance access and monitoring of a critically ill patient; it should also contain an automated or sensor-based operated sink. If a single room was not possible and two patients or more are needed in one room, we should keep a minimum 2.4-meter distance between beds and adhere to all infection control standards. Ideally, every single large room should have its own bathroom; however, a designated one patients’ bathroom in the unit should suffice provided a strict adherence to all infection control measures to prevent cross-contamination. We should have one single-isolation negative-pressure room for each six-patient room with its own bathroom and ante-room [16]. Technically, clinical rooms are not patients’ rooms but are linked to clinical services like storage of large equipment, clean linen, and soiled linen. These rooms should be located near to PICU, shouldn’t be mixed, and should be clearly designated for infection control reasons as well as for the national and international accreditation requirements. Non-clinical rooms like staff lounge, staff offices, on-call rooms, conference room, and family counseling room must be in close proximity to the PICU unit to facilitate traffic without compromising patient care [16,19].

## Staff

The staff here means the manpower needed to operate PICU and meet the required standards. The number of required staff in PICU is a moving target that depends on PICU level (level I Vs. Level II), the number of beds, PICU staff activity outside PICU (like rapid response team (RRT), transport or procedural sedation coverage). At a minimum, we need a designated medical director for PICU as well as an assigned head nurse with allocated offices and secretarial support [1,16,18,19]. The medical director should be a certified intensivist with mixed clinical and administrative duties and should be available to PICU 24/7. Other medical staff should be available in numbers that maintain the workflow and satisfy regulations for working hours per week based on the composition of the medical team and the level of PICU (PICU physician, PICU fellow, PICU assistant consultant, nurse practitioner). Monitoring and direct observation of critically ill patients is the core service in PICU and despite the advanced technology in invasive and non-invasive monitoring in critical care, direct human monitoring (by nurses) cannot be replaced by any technology. Therefore, nurses are the cornerstone of a dynamic PICU; hence, nurse: patient ratio is also dynamic and should cover a range from 2:1 (for complex patients on ECMO) to 1:2 (for stable chronically ill patients prior to discharge from PICU) with charge nurse (not assigned to any patient) to oversees the bedside care and supports junior staff (1,16,18,19). Respiratory therapist (RT) is another pillar of PICU, and the PICU staffing plan should include them with a ratio of 1 RT for each of four ventilated patients [1,18]. In modern PICUs, dietitians, clinical pharmacists, and physiotherapists are essential PICU staff members with specific responsibilities like a daily round in PICU at least in level I PICU, where their presence is linked to positive outcomes like decreasing LOS, HAI and preventing post-ICU syndromes [18].

## Stuff

The stuff here means all equipment, supply, and machines that are needed to operate the unit. We can divide them into general and specific stuff where general stuff is fixed items for all PICUs, and specific stuff is more case-based or per bed requirements.

### For general stuff:

All critical care should have medical gases (compressed gases) that include a minimum of two O2 outlets, two medical air, and two vacuum outlets per bed. Electricity is a delicate issue in critical care where each bed should have a minimum of 10-12 points, including 50% of them as uninterrupted power source (UPS) that’s connected directly to the emergency backup generator; furthermore, the level of these points preferably should be at least one meter above the ground for easy access [16,19]. We need a central station for each unit (or pod) with an audio and visual feedback alarm system with the capability of recording and retrieving services. Beds size and characteristics are important to be tailored to the PICU population, where usually around 25% are cribs (with side rails), and the remaining are regular (adult size) beds with scale capability. In PICU that admits neonates and ex-preemies less than 2 K.G., they need to have extra incubators with the capability for temperature regulation and movable parts for procedures and resuscitation proposes. Cardiopulmonary monitoring devices and screens are essential to have for each bed with the capability of projecting continuous vital signs readings in different colors. Crash-cart is a mandatory requirement for each unit (or POD) where it should be located inside the unit in a designated place and contains all essential resuscitation equipment, supply, and medication labeled and prioritized. Medical Waste containers, sharp containers, and trash pins should be available and distributed in the unit as per infection control recommendations. Computers (fixed P.C.s) and computer on wheels (COWS) are an integral part of modern paperless hospitals and should be available in enough quantity for all PICU staff to facilitate their daily work, including rounds and documentation. Blood gas machines are now a standard bedside (point of care) service in every critical care unit where blood gas samples can be analyzed within a short period as well as provide extra helpful lab value like co-oximetry, essential electrolytes, and lactate. It’s preferable to have a portable x-ray machine dedicated for PICU near PICU for easy access and infection control purposes. The presence of a computer-controlled medication dispensing machine in PICU will create easy access for the common drugs used in PICU and facilitate drug delivery during a crisis in addition to the satellite pharmacy.

### For specific stuff:

The respiratory system requirements are a priority given that it’s the most frequent system affected and compromised in PICU with the need for extensive equipment and supplies. Conventional Ventilators (invasive and non-invasive) are the core equipment for respiratory support where most patients are in need of either invasive or non-invasive ventilators with pediatric specifications. Pediatric specification, type of modes available, minimum tidal volume (Vt), circuits, and humidifiers are best discussed beforehand with a senior R.T. with pediatric experience. Non-conventional ventilators like HFOV are important rescue interventions. Ideally, they should be available for a wide range of pediatric ages and weight, i.e., HFOV 3100-A for pediatrics with less than 30 kg and 3100-B for a pediatric patient weighing more than 30 Kg. The Nitric oxide delivery system is another critical rescue machine for iNO that should be available in PICU along with its gas supply. Extra Ventilators that are designed for transport are essential in PICU and preferably also MRI-compatible ventilators. Airway equipment and supply are usually reviewed with R.T. in terms of quantity and quality to match the patients’ complexity and population, which include: ETTs, capnography devices, oral/nasal airway devices, and laryngoscopes as well as a set for difficult airway (LMAs, video-laryngoscope, bronchoscope, cricothyrotomy set). Central venous lines and arterial lines are used frequently in PICU and need to be available in all different sizes to accommodate different ages and sizes in PICU; furthermore, we sometimes utilize these lines also for invasive monitoring, i.e., B.P., CVP which requires a special device (transducer) to achieve this goal, however, it needs to be calibrated and compatible with another monitoring system. Currently, most central lines are inserted under the ultrasound (U.S.) guide, which leads to less traumatic insertion, less infection, and shorter time; however, the U.S. should be accompanied by pediatric size probes with different functions. Fluid and drug delivery systems are an integral part of daily PICU care. They should be available in sufficient amounts to support critically ill patients in the form of infusion pumps, syringe pumps, and feeding pumps; however, new smart pumps are also available with dual actions.

## Discussion

Establishing a new PICU is challenging for administrators, leaders, project managers, and intensivists who participate in this complex process. Most hospitals in the commissioning phase of establishing a new hospital utilize a pediatric intensivist to be part of the establishing team or even the team leader to create and design a modern PICU; hence, the need for the intensivist to be well-versed in this topic.

The intensivist perspective in establishing a new PICU is different and sometimes opposing to the perspective of the administrator or the project manager, where the former approach is from a patient-staff-centered view and the latter approach is from a cost-benefit, budget, and macrosystem integration point of view. An intensivist, well-versed fundamentals in establishing new PICU, will be able to reconcile these -seemingly- two opposing points of view and create a modern, cost-effective, patient-centered and staff-centered PICU that integrates well in the macrosystem level of the critical care services at either hospital level or even at the regional level. The intensivist should bring the attention of the team to the known impact of PICU design on patients on PICU staff like the location of PICU unit, size of the unit, size of the patient’s rooms, the internal design of patient’s room, location of clinical and non-clinical rooms and traffic control design and measures in PICU. However, we should know and appreciate the constraints on administrators and leaders regarding budget and other competing priorities knowing that establishing a new PICU is only one small part of the whole project of commissioning and operating a new pediatric hospital.

In this paper, we introduced a new mental framework to provide the pediatric intensivist with a high-level view or bird’s view about the essential requirements to establish a new PICU and to be an effective team member or leader in the efforts of creating a healing environment for critically ill pediatric. We use the abbreviation (4S) to donate to the four major aspects in the process of establishing a new PICU which include: System, space, staff, and stuff. Such a holistic approach will ensure establishing a patient-centered PICU that meets the minimal requirement for national and international accreditation entities.

Finally, we need to be cognizant of and acknowledge the dynamic interaction between these 4S, where the available space can limit the stuff and the available qualified staff could impact the space and the stuff. Moreover, these components are better to be visualized as complementary to each other rather than separate or independent components.

## Conclusion

The 4S conceptual framework (System, Space, Staff, and Stuff) is an easy tool for intensivist to use in preparedness for establishing a new PICU. It’s not a replacement for the due diligence of detailed planning, rather it’s a guide and should be used in that context.

## Supplementary Material

1

## Figures and Tables

**Table 1:** Cheat-sheet for 4S framework.

Category:	Item name	Category	Item name
**1- System**	Organization chart	**4- Stuff**	**General:**
	Scope of service		Beds: adult sizes with air mattress (25% cribs), capability for weighing
	Admission and discharge criteria		Compressed gases (min 2 O2, 2 air 2 suction) per beds
	Guidelines for common disease (preferred as manual including common drug doses, routes and concertation)		Electrical points min 12 per bed (including UPS)
	clinical pathway (minimum 2) i.e. DKA, Status epilepticus		Central station (with capablity for recording and retrieving)
	Protocol (like sedation, weaning from ventilation, early rehibition)		Crash cart with defibrillator / CPR board (preferable with audio-visual feedback)
	A job description for PICU staff i.e. consultant, specialist / assistant consultant, resident..		Mobile x-ray machine
	a set of clinical indicators and KPI like: morality, LOS, occupancy rate, readmission rate		Cardiopulmonary monitoring for each bed
	Visitation guidelines, family presence/sleepover, sitters/watchers). (written as P&P)		PCs, COWS, printer, scanner, clocks (for patient orientation)
**2- space:**	Location near to EM, OR		Waste pins (medical and nonmedical)
	Min 6 beds max 14 beds (if more than 14 >> pods)		Computer-controlled safe for dispensing common drugs
	Preferred single room with bathroom, sink /basin (sensor based control)		**Specific:**
	If more than one patients in one room: 2.4 M distance between beds.		Cooling system with temp probs
	Negative pressure isolation room (1 for each 6 beds).		Ventilator (invasive and noninvasive) + INO machine
	Rooms for medical equipment storage, clean Lenin and soiled Lenin		Transport ventilator
	Rooms for on-calls, family lounge, family counselling		Airway equipment (ETTs, airway devices, laryngoscope, bronchoscope, suction devices,) + difficult airway equipment, capnography
	Medical offices and education room/conference rooms		Bronchoscope
	Staff restrooms and lounges (preferably separate male and female)		Blood gas machine
**3- staff**	medical director (responsible for overall PICU outcome)		Infusion pumps / syringe pumps
	Head nurse (dedicated for PICU)		NGTs / GTs / colostomy bags
	qualified Intensivists (min 3 +/− part-timers)		Foley’s catheter, urinometer, urinal, commode (chairs)
	PICU specialist /assistant staff /nurse practitioners (min 5 to meet the required hours)		US machine with probes and sterile cover/sterile gel
	Qualified critical care Nurses min 1:1 PLUS charge nurse (3.5 - 4 Per bed)		Central lines (different sizes)
	RTs (1 for each 4 ventilated beds) or 1 for each 10 beds		Staff communication system i.e. Vocera
	Qualified Clinical pharmacist		Handover system i.e. iPass
	Physiotherapist		CRRT
	Qualified clinical dietitian		Ventilator MRI-compatible
	Play therapist		Infusion pumps MRI-compatible
	Clerks		White board (for daily tasks)

## Data Availability

No data set were produced by this work.

## Abbreviations:

R.T.: Respiratory therapist
R.N.: Registered nurse
PICU: Pediatric intensive care unit
NICU: Neonatal intensive care unit
JCI: Joint commission international for hospital standards
CBAHI: the Saudi Central board for Accreditation for healthcare institutions
E.D.: Emergency department
OR: Operation theater
