# Supplementary material for: System, Space, Staff, and Stuff framework in establishing a new pediatric critical care unit (PICU) (4S Framework)
Source: J Pediatr Perinatol Child Health. Author manuscript; Available in PMC 2022 Nov 16. (PMC9668042; doi:10.26502/jppch.74050129)
Supplement: 1 [file NIHMS1846299-supplement-1.pdf]

Supplement 1: Minimum Guidelines and Levels of Care for PICUs.

Item:	Level I	Level II
**I. Organization and administrative structure**		
**A. Category I facility**	E	E
**B. Organization**		
1. PICU committee	E	E
2.Distinct administrative unit	E	E
3.Delineation of physician and nonphysician privilege	E	E
**C. Policies**		
1. Admission and discharge	E	E
2. Patient monitoring	E	E
3. Safety	E	E
4. Nosocomial infection	E	E
5. Patient isolation	E	E
6. Family-centered care	E	E
7. Traffic control	E	E

8. Equipment maintenance	E	E
9. Essential equipment breakdown	E	E
10. System of record keeping	E	E
11. Periodic review		
a. Morbidity and mortality	E	E
b. Quality of care	E	E
c. Safety	E	E
d. Critical care consultation	E	E
e. Long-term outcomes	D	D
f. Supportive care	D	D
**D. Physical facility—external**		
1. Distinct, separate unit	E	D
2. Distinct unit (not necessarily physically separate) with auditory and visual separation	E	E
3. Controlled access (no through-traffic)	E	E
4. Located near:		
a. Elevators	E	D
b. Operating room	D	D
c. Emergency room	D	D
d. Recovery room	D	D
e. Physician on-call room	E	D
f. Nurse manager's office	D	D
g. Medical director's office	D	D
h. Waiting room	E	D
5. Separate rooms available:		
a. Family counseling room	E	D
b. Conference room	D	D
c. Staff lounge	D	D
d. Staff locker room	D	D
e. Storage lockers for patients' personal effects (may be internal)	E	E
f. Family sleep area and shower	E	D
**E. Physical facility—internal**		
1. Patient isolation capacity	E	E
2. Patient privacy provision	E	E

3. Satellite pharmacy	D	O
4. Medication station with drug refrigerator and locked narcotics cabinet	E	E
5. Emergency equipment storage	E	E
6. Clean utility (linen) room	E	E
7. Soiled utility (linen) room	E	E
8. Nourishment station	E	E
9. Counter and cabinet space	E	E
10 .Staff toilet	E	E
11. Patient toilet	E	E
12. Hand-washing facility	E	E
13. Clocks	E	E
14. Televisions, radios, toys	E	E
15. Easy, rapid access to head of bed	E	E
16. 12 or more electrical outlets per bed	E	E
17. 2 or more oxygen outlets per bed	E	E
18. 2 or more compressed air outlets per bed	E	E
19. 2 vacuum outlets per bed	E	E
20. Computerized laboratory reporting or efficient equivalent	E	D
21. Building code or federal code conforming for:		
a. Heating, ventilation, and air conditioning	E	E
b. Fire safety	E	E
c. Electrical grounding	E	E
d. Plumbing	E	E
e. Illumination	E	E
**II. Personnel**		
**A. Medical director**		
1. Appointed by appropriate hospital authority and acknowledged in writing	E	E
2. Qualifications		
a. Board certified or actively pursuing certification in 1 of the following:		
i. Pediatric critical care medicine	E	E
• Initial board certification in pediatrics	E	E
• Codirector if director is not a pediatrician	E	D

ii. Anesthesiology with practice limited to infants and children and special qualifications in critical care medicine	E	E
iii. Pediatric surgery with added qualification in surgical critical care medicine	E	E
3. Responsibilities documented in writing	E	E
a. Acts as primary attending physician	D	D
b. Has authority to provide consultation when physician is not available	E	E
c. Assumes patient care if primary attending physician is not available	E	E
d. Participates in development, review, and implementation of PICU policies*	E	E
e. Maintenance of database and/or vital statistics*	E	E
f. Supervises quality-control and quality-assessment activities (including morbidity and mortality reviews)*	E	E
g. Supervises resuscitation techniques (including educational component)*	E	E
h. Ensures policy implementation*	E	E
i. Coordinates staff education*	E	E
j. Participates in budget preparation*	E	E
k. Coordinates research*	E	D
4. Substitute physician available to act as attending physician in medical director's absence	E	E
**B. Physician staff**		
1. A physician in-house 24 h per day	E	E
a. A physician at the postgraduate year 2 level or above assigned to the PICU	E	D
b. A physician at the postgraduate year 2 level or above available to the PICU (advanced practice nurse or physician assistant may be used)	E	E
c. A physician at the postgraduate year 3 level or above (in pediatrics or anesthesiology) in-house 24 h per day	E	O
2. Available in 30 min or less (24 h per day)		
a. Pediatric intensivist or equivalent	E	D
3. Available in 1 h or less		

a. Anesthesiologist	E	E
i. Pediatric anesthesiologist	E	D
b. General surgeon	E	E
c. Surgical subspecialists		
i. Pediatric surgeon	E	D
ii. Cardiovascular surgeon	E	O
• Pediatric cardiovascular surgeon	D	O
iii. Neurosurgeon	E	E
• Pediatric neurosurgeon	E	O
iv. Otolaryngologist	E	D
• Pediatric otolaryngologist	D	O
v. Orthopedic surgeon	E	D
• Pediatric orthopedic surgeon	D	O
vi. Craniofacial, oral surgeon	D	O
4. Pediatric subspecialists:		
a. Intensivist	E	E
b. Cardiologist	E	D
c. Nephrologist	E	D
d. Hematologist/oncologist	D	D
e. Pulmonologist	D	D
f. Endocrinologist	D	D
g. Gastroenterologist	D	D
h. Allergist	D	D
i. Neonatologist	E	E
j. Neurologist	E	D
k. Geneticist	D	D
5. Radiologist	E	E
a. Pediatric radiologist	E	O
6. Psychiatrist or psychologist	E	D
**C. Nursing staff**		
1. Manager/director	E	E
a. Training and clinical experience in pediatric critical care	E	E
b. Master's degree in pediatric nursing or nursing administration	D	D

2. Nurse-to-patient ratio based on patient need	E	E
3. Nursing policies and procedures in place	E	E
4. Orientation to PICU	E	E
5. Completion of clinical and didactic critical care course	E	E
6. Address psychosocial needs of patient and family	E	E
7. Participate in continuing education	E	E
8. Completion of critical care registered nurse (pediatric) certification	D	D
9. Completion of PALS or an equivalent course	D	D
10. Nurse educator on staff (clinical nurse specialist)	E	D
a. Responsible for pediatric critical care in-service education	E	D
11. Nurse coordinator for regional continuing education	O	O
**D. Respiratory therapy staff**		
1. Supervisor responsible for training registered respiratory therapy staff	E	E
2. Maintenance of equipment and quality control and review	E	E
3. Respiratory therapist in-house 24 h per day assigned primarily to PICU	E	D
4. Respiratory therapist in-house 24 h per day	E	E
5. Respiratory therapists familiar with management of pediatric patients with respiratory failure	E	E
6. Respiratory therapists competent with pediatric mechanical ventilators	E	E
7. Completion of PALS or an equivalent course	D	D
**E. Other team members**		
1. Biomedical technician (in-hospital or available within 1 h, 24 h per day)	E	E
2. Unit clerk on staff 24 h per day with a written job description	E	D
3. Child life specialist	E	D
4. Clergy	E	E
5. Social worker	E	E
6. Nutritionist or clinical dietitian	E	E
7. Physical therapist	E	E
8. Occupational therapist	E	E

9. Pharmacist (24 h per day)	E	E
10. Pediatric clinical pharmacist	D	D
11. Radiology technician	E	E
12. Bereavement coordinator	D	D
**III. Hospital facilities and services**		
A. Emergency department		
1. Covered entrance	E	E
2. Separate entrance	E	D
3. Adjacent helipad	D	D
4. Staffed by physician 24 h per day	E	E
a. Trained in pediatric emergency medicine	D	D
5. Resuscitation area		
a. 2 or more areas with capacity and equipment to resuscitate medical, surgical, and trauma pediatric patients	E	D
b. 1 or more areas as described above	E	E
B. Intermediate care unit or step-down unit separate from PICU and pediatric acute care unit	D	D
C. Pediatric rehabilitation unit	D	D
D. Blood bank		
1. Comprehensive (all blood components)	E	E
2. Type and cross match within 1 h	E	E
E. Radiology services and nuclear medicine		
1. Portable radiograph	E	E
2. Fluoroscopy	E	D
3. Computed tomography scan	E	E
4. Magnetic resonance imaging	E	D
5. Ultrasound	E	E
6. Angiography	E	O
7. Nuclear scanning	E	O
8. Radiation therapy	D	O
F. Laboratory with microspecimen capability:		
1. Available within 15 min		
a. Blood gases	E	E
2. Available within 1 h		
a. Complete blood cell, platelet, and differential counts	E	E

b. Urinalysis	E	E
c. Chemistry profile (electrolytes, serum urea nitrogen, glucose, calcium, and creatinine)	E	E
d. Clotting studies	E	E
e. Cerebrospinal fluid analysis	E	E
3. Available within 3 h:		
a. Ammonia concentration	E	E
b. Drug screening	E	E
c. Osmolality	E	E
d. Magnesium and phosphorus concentrations	E	E
e. Toxicology screen	E	D
4. Preparation available 24 h per day		
a. Bacteriology (culture and Gram-stain)	E	E
5. Point-of-care diagnostic testing	D	D
G. Department of surgery		
1. Operating room available within 30 min, 24 h per day	E	E
2. Second operating room available within 45 min, 24 h per day	E	D
3. Capabilities:		
a. Cardiopulmonary bypass	E	D
b. Bronchoscopy (pediatric)	E	D
c. Endoscopy (pediatric)	E	D
d. Radiograph in operating room	E	E
H. Cardiology department with pediatric capability		
1. Electrocardiography	E	E
2. Echocardiography		
a. Two-dimensional echocardiography with Doppler	E	E
3. Catheterization laboratory (pediatric)	D	O
I. Neurodiagnostic laboratory		
1. EEG	E	E
2. Evoked potentials	D	D
3. Transcranial Doppler flow	D	O
J. Hemodialysis	E	O
K. Peritoneal dialysis or continuous renal replacement therapy	E	O

L. Pharmacy with pediatric capability	E	E
1. Available 24 h per day for all requests	E	E
2. Located near PICU and pediatric acute care unit	D	O
3. Urgent drug-dosage form at bedside	E	E
4. Satellite pharmacy located in PICU	D	O
5. Pediatric pharmacist available for medical rounds	D	O
M. Rehabilitation department with pediatric capability		
1. Physical therapy	E	E
2. Speech therapy	E	E
3. Occupational therapy	E	E
IV. Drugs and equipment		
A. Emergency drugs	E	E
B. Portable equipment:		
1. Emergency cart	E	E
2. Procedure lamp	E	E
3. Doppler ultrasonography device	E	E
4. Infusion pumps (with microinfusion capability)	E	E
5. Defibrillator and cardioverter	E	E
6. Electrocardiography machine	E	E
7. Suction machine (in addition to bedside)	E	E
8. Thermometers	E	E
9. Expanded scale electronic thermometer	E	E
10. Automated blood pressure apparatus	E	E
11. Otoscope and ophthalmoscope	E	E
12. Automatic bed scale	E	D
13. Patient scales	E	E
14. Cribs (with head access)	E	E
15. Beds (with head access)	E	E
16. Infant warmers, incubators	E	E
17. Heating and cooling blankets	E	E
18. Bilirubin lights	E	E
19. Transport monitor	E	D
20. EEG machine	E	E
21. Isolation cart	E	E

22. Blood warmer	E	E
23. Pacer (transthoracic or transvenous)	E	E
C. Small equipment:		
1. Tracheal intubation equipment	E	E
2. Endotracheal tubes (all pediatric sizes)	E	E
3. Oropharyngeal and nasopharyngeal airways	E	E
4. Vascular access equipment	E	E
5. Cut-down trays	E	E
6. Tracheostomy tray	E	E
7. Flexible bronchoscope	E	D
8. Cricothyroidotomy tray	E	E
D. Respiratory support equipment		
1. Bag-valve-mask resuscitation devices	E	E
2. Oxygen tanks	E	E
3. Respiratory gas humidifiers	E	E
4. Air compressor	E	E
5. Air-oxygen blenders	E	E
6. Ventilators of all sizes for pediatric patients	E	E
7. Inhalation therapy equipment	E	E
8. Chest physiotherapy and suctioning	E	E
9. Spirometers	E	E
10. Continuous oxygen analyzers with alarms	E	E
E. Monitoring equipment:		
1. Capability of continuous monitoring of:		
a. Electrocardiography, heart rate	E	E
b. Respiration	E	E
c. Temperature	E	E
d. Systemic arterial pressure	E	E
e. Central venous pressure	E	E
f. Pulmonary arterial pressure	E	D
g. Intracranial pressure	E	D
h. Esophageal pressure	D	O
i. Capability to measure 4 pressures simultaneously	E	D
j. Capability to measure 5 pressures simultaneously	D	D

k. Arrhythmia detection and alarm	E	E
l. Pulse oximetry	E	E
m. End-tidal CO_2	E	E
2. Monitor characteristics:		
a. Visible and audible high and low alarms for heart rate, respiratory rate, and all pressures	E	E
b. Hard-copy capability	E	E
c. Routine testing and maintenance	E	E
d. Patient isolation	E	E
e. Central station	E	E
V. Prehospital care		
A. Integration and communication with EMS system	E	E
B. Transfer arrangements with referral hospital	E	E
C. Transfer arrangement with level I PICU	NA	E
D. Educational programs in stabilization and transportation for EMS personnel	E	D
E. Transport system (including transport team)	E	O
F. Emergency communication into PICU and pediatric acute care unit (eg, phone, radio) 24 h per day	E	E
G. Communication link to poison control center	E	E
VI. Quality improvement		
1. Collaborative quality assessment	E	E
2. Morbidity and mortality review	E	E
3. Utilization review	E	E
4. Medical records review	E	E
5. Discharge criteria (planning)	E	E
6. Safety review	E	E
7. Long-term follow-up of patients and family	D	D
VII. Training and continuing education		
A. Physician training		
1. Unit in facility with accredited pediatric residency program	D	O
2. Unit provides clinical rotation for pediatric residents in pediatric critical care	D	O
3. Fellowship program in pediatric critical care	D	O
4. Cardiopulmonary resuscitation certification	E	E

5. PALS or advanced pediatric life support	E	E
6. Ongoing continuing medical education for physicians specific to pediatric critical care	E	E
7. Staff physicians to attend and participate in pediatric critical care	E	E
B. Unit personnel:		
1. Cardiopulmonary resuscitation certification for nurses and respiratory therapists	E	E
2. Resuscitation practice sessions	E	E
3. Ongoing continuing education (on-site and/or off-site workshops and programs for nurses respiratory therapists, clinical pharmacists)	E	E
4. Certified by the American Association of Critical Care Nurses	D	D
5. PALS or advanced pediatric life support certification	E	E
6. Critical care registered nurse certification	D	D
C. Regional education		
1. Participation in regional pediatric critical care education	E	O
2. Service as educational resource center for public education in pediatric critical care	D	D
3. Prehospital care and interhospital transport	D	O

- Footnote:
- E indicates essential; D, desired; O, optional; NA, not applicable.
- (curtesy of David I. Rosenberg et al)

Supplement 1: Requirements of AAP/SCCM for qualification in PICU (see reference 1 table supp).
